# Infection Route Impacts the Pathogenesis of Severe Fever with Thrombocytopenia Syndrome Virus in Ferrets

**DOI:** 10.3390/v14061184

**Published:** 2022-05-29

**Authors:** Su-Jin Park, Young-Il Kim, Mark Anthony Casel, Eun-Ha Kim, Se-Mi Kim, Kwang-Min Yu, Rare Rollon, Seung-Gyu Jang, Hye Won Jeong, Young Ki Choi

**Affiliations:** 1 Division of Life Science and Research Institute of Life Science, Gyeongsang National University, Jinju 52828, Korea; 2 Center for Study of Emerging and Re-emerging Viruses, Korea Virus Research Institute, Institute for Basic Science (IBS), Daejeon 34126, Korea; kyibb@chungbuk.ac.kr (Y.-I.K.); mbcasel@up.edu.ph (M.A.C.); semikim@ibs.re.kr (S.-M.K.); rarerollon@gmail.com (R.R.); sgjang94@chungbuk.ac.kr (S.-G.J.); 3 College of Medicine and Medical Research Institute, Chungbuk National University, Cheongju 28644, Korea; dealereh@hanmail.net (E.-H.K.); dbrhkdals7@gmail.com (K.-M.Y.); hwjeong@chungbuk.ac.kr (H.W.J.); 4 Zoonotic Infectious Diseases Research Center, Chungbuk National University, Cheongju 28644, Korea

**Keywords:** SFTSV, animal model, aged ferret, infection routes

## Abstract

The threat of severe fever with thrombocytopenia syndrome (SFTS) to public health has been increasing due to the rapid spread of the ticks that carry the causative viral agent. The SFTS virus (SFTSV) was first identified in China and subsequently detected in neighboring countries, including South Korea, Japan, and Vietnam. In addition to the tick-mediated infection, human-to-human transmission has been recently reported with a high mortality rate; however, differential study of the pathogen has been limited by the route of infection. In this study, we investigated the pathogenic potential of SFTSV based on the infection route in aged ferrets, which show clinical signs similar to that of human infections. Ferrets inoculated with SFTSV via the intramuscular and subcutaneous routes show clinical signs comparable to those of severe human infections, with a mortality rate of 100%. Contrastingly, intravascularly infected ferrets exhibit a comparatively lower mortality rate of 25%, although their early clinical signs are similar to those observed following infection via the other routes. These results indicate that the infection route could influence the onset of SFTS symptoms and the pathogenicity of SFTSV. Thus, infection route should be considered in future studies on the pathogenesis of SFTSV infection.

## 1. Introduction

Severe fever with thrombocytopenia syndrome (SFTS) is caused by the SFTS virus (SFTSV) belonging to the genus *Bandavirus* (family *Phenuiviridae*, order *Bunyavirales*). The major clinical manifestations of SFTSV infection are fever, thrombocytopenia, leukopenia, and elevated serum hepatic enzymes [1]. The first report of SFTSV was in China in 2009, after which it was identified in South Korea, Japan, and Vietnam [2,3,4,5]. The age of patients with SFTSV infection ranges from 2 months (suspected cases) to 100 years, with fatality rates varying geographically from 1.6% to 30% [6,7]. However, the majority of cases have been reported in people over 40–50 years old, and the number of fatal cases dramatically increases in those over 60 [6,7,8].

SFTSV is maintained in nature through tick-to-vertebrate cycles [9]. Although SFTSV infection is predominantly transmitted through tick bites, human-to-human and cat-to-human transmissions have also reportedly occurred through direct contact or exposure to bodily fluids [10,11].

Species of ticks identified as SFTSV vectors include *Haemaphysalis longicornis*, *Rhipicephalus microplus*, *Haemaphysalis flava*, *Haemaphysalis concinna*, *Amblyomma testudinarium*, and *Ixodes nipponensis* [12,13,14]. *H. longicornis* is mainly found in eastern Asia, Australia, New Zealand, and the eastern United States [15]. There is potential for global distribution of the virus due to expansion of the tick habitat caused by climate change, and the extensive range of mammalian to avian hosts [15,16]. Therefore, SFTSV was listed in the World Health Organization of Research and Development Blueprint in 2018 as a pathogen with potential to cause a public health emergency [17].

SFTSV has a wide range of hosts, including animals and humans [18]. However, suitable animal models for SFTSV infection, which show a high mortality similar to that observed in human infection cases, are quite limited. Thus, there is an urgent need to develop SFTSV-susceptible animal models to increase understanding of the detailed pathogenesis mechanisms of this virus and to develop countermeasures against SFTSV infections. Mice, rhesus macaque, and cynomolgus macaques showed non-fatal outcomes with only mild to no disease symptoms following infection with SFTSV [19,20]. Mitomycin-treated mice, interferon receptor-deficient (IFNAR-/-) mice, newborn mice, and humanized NOD-Prkdc*^em26Cd52^*Il2rg*^em26Cd22^*/Nju mice are susceptible to SFTSV infection with high fatality rates [21,22,23,24]. However, there are difficulties associated with using these immunocompromised animals to study the pathogenesis of the infection and to evaluate the efficacy of antivirals, which may require proper immune responses. In recent studies in aged ferrets with intact immunologic function, SFTSV infection via intramuscular (IM) injection resulted in severe clinical symptoms, including acute fever, high viral load, thrombocytopenia, leukopenia, and hematological changes; fatalities were also recorded [8,25]. However, because tick bites, which are the natural infection route, reportedly induce subcutaneous (SC) infections, the best route for infection remains uncertain.

In this study, we compared susceptibility and disease outcomes of SFTSV infection in aged ferrets with different routes of infection. SFTSV was administered via IM, SC, and intravenous (IV) routes, and the results show that ferrets are susceptible to SFTSV infection via the SC and IM routes. Additionally, infection through the IM and SC routes results in severe clinical symptoms and higher mortality rates compared to the IV infection route.

## 2. Materials and Methods

### 2.1. Study Approval

All animal experiments were approved by the Medical Research Institute, a member of the Laboratory Animal Research Center of Chungbuk National University (LARC) (approval number: CBNUA-1539-21-01), and were conducted in strict accordance and adherence to relevant policies, as mandated under the Guidelines for Animal Use and Care of the Korea Centers for Disease Control (K-CDC) in an enhanced biosafety level 3 (BSL3) containment laboratory.

### 2.2. Virus and Cells

Stocks of CB1/2014 SFTSV were propagated on confluent monolayers of Vero E6 cells (ATCC No. CRL-1586; American Type Culture Collection, Manassas, VA, USA) by infecting at 0.01 multiplicity of infection. The supernatant was collected seven days post-infection (dpi) for analysis. Viral titers were determined through an immunostaining assay in Vero E6 cells. Briefly, confluent monolayers of Vero E6 cells were infected with a serially diluted virus in 1% fetal bovine serum (FBS), followed by incubation for 1 h at 37 °C. An overlay medium containing a final concentration of 0.8% agarose in 1% FBS-Dulbecco’s Modified Eagle’s Medium was then added to the cells. Next, the cells were incubated for six days before fixation, incubated in an in-house generated monoclonal nucleoprotein antibody against SFTSV, and visualized with 3,3’-diaminobenzidine staining [25].

### 2.3. Infection of Ferrets

Groups of SFTSV antibody-free, female ferrets (age, 3–4 years old; n = 8 per group) were inoculated with CB1/2014 (1 × 10^5^ focus forming unit (FFU)/mL) via the three different routes. In addition, a mock group of female ferrets (age, 3–4 years old; n = 8 per group) were intramuscularly inoculated with phosphate-buffered saline (PBS). The virus was injected into both thighs (0.5 mL), over the skin of the left shoulder, or into the jugular vein of ferrets in the IM, SC, and IV groups, respectively.

### 2.4. Observation of Clinical Signs

The survival was monitored for 14 days after infection. Clinical signs (body weight, body temperature, survival, hematological changes) were monitored every other day for 14 days post-infection (dpi). Platelets and white blood cells (WBCs) were analyzed using a Celltac hematology analyzer (MEK-6550J/K; Nihon Kohden, Tokyo, Japan). The analysis was performed on ethylenediaminetetraacetic acid-treated whole-blood samples. The levels of alanine aminotransferase (ALT) and aspartate aminotransferase (AST) in serum samples were analyzed using a Celltac alpha analyzer (MEK-6550, Nihon Kohden).

### 2.5. Viral Copy Numbers by Reverse Transcription-Quantitative Polymerase Chain Reaction (RT-qPCR)

Serum were collected from the PBS-inoculated (mock) and virus-infected groups at 2-day intervals after infection. To evaluate viral replication, groups of ferrets (n = 3) were sacrificed at 6 dpi, and spleen, liver, kidney, intestine, and brain tissues were collected. Total RNA was extracted from serum samples using TRIzol LS Reagent (Thermo Fisher Scientific, Waltham, MA, USA), after which cDNA was generated by reverse transcription using a QuantiTect Reverse Transcription kit (QIAGEN, Hilden, Germany). Real-time polymerase chain reaction was performed using SYBR Green Supermix (Bio-Rad Laboratories, Inc., Hercules, CA, USA) and a CFX96 real-time PCR detection system (Bio-Rad). Viral copy numbers were determined by RT-qPCR using an S segment-based SFTSV-specific primer set: forward primer, SFTSV-S-F, gcagttggaatcaggga; and reverse primer, SFTSV-S-R, cccacttggacatgtgct. The copy number was calculated as a ratio to the standard control.

### 2.6. Histopathology

Spleen and liver tissues were harvested at 6 dpi from each group, and samples were fixed in 10% neutral-buffered formalin and embedded in paraffin. Histological assessment was conducted using standard hematoxylin and eosin staining and light microscopy (magnification x100). The slides were viewed using an Olympus IX 71 (Olympus, Tokyo, Japan) microscope and DP controller software to capture images.

### 2.7. Statistical Analyses

Statistical analyses were performed using Prism software 9.3.1 (GraphPad Software Inc., La Jolla, CA, USA). Two-way ANOVA with Tukey’s comparison test or Kaplan–Meier survival curves with a log-rank (Mantel–Cox) test was used. An asterisk (*) indicates statistical significance when data are compared to those from the mock group (*p < 0.05; ** *p* < 0.01; *** *p* < 0.001; and **** *p* < 0.0001). A section sign (§) indicates statistical significance when data are compared among the virus-infected groups (§ *p* < 0.05, §§ *p* < 0.01, §§§ *p* < 0.001, §§§§ *p* < 0.0001) with respect to viral copy numbers in serum and tissues.

## 3. Results

### 3.1. Variation in Mortality Rate with Infection Route

Aged ferrets were infected with CB1/2014 SFTSV via the IM, SC, and IV routes to compare the susceptibility to infection. The mock group was comprised of ferrets (n = 8) injected with PBS by the IM route. Temperature, body weight, hematological changes, and survival were monitored in all groups for 14 days.

**Figure 1:**
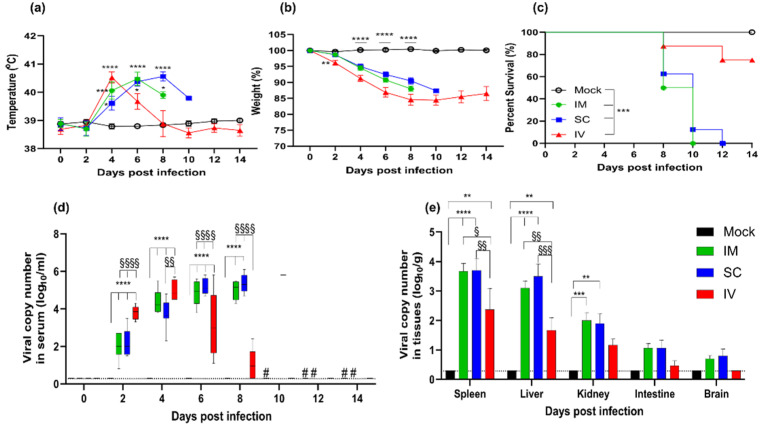
Virulence of severe fever with thrombocytopenia syndrome virus (SFTSV) in aged ferrets via different routes of infection. Eight ferrets per group were inoculated with SFTSV via the intramuscular (IM), subcutaneous (SC), or intravenous (IV) route. Animals were then assessed every other day for (**a**) temperature changes; (**b**) relative weight; (**c**) Kaplan–Meier survival; (**d**) and viral copy numbers in serum. (**e**) Viral copy numbers in spleen, liver, kidney, intestine, and brain were examined at 6 dpi. The data for the mock, IM, SC, and IV groups are shown in black, green, blue, and red, respectively. Data are presented as mean ± s.e.m (**a**,**b**,**e**) or min to max (**d**), and titers below the limit of detection are shown as 0.3 log_10_ RNA copy numbers/mL or 0.3 log_10_ RNA copy numbers/g (dashed lines). An asterisk (*) indicates statistical significance when data are compared to those for the mock group (* *p* < 0.05; ** *p* < 0.01; *** *p* < 0.001; and **** *p* < 0.0001). A section sign (§) indicates statistical significance when data are compared among the virus-infected groups (§ *p* < 0.05, §§ *p* < 0.01, §§§ *p* < 0.001, §§§§ *p* < 0.0001) with respect to viral copy numbers in serum. Statistical significance was determined by two-way ANOVA with Tukey’s comparison test (**a**,**b**,**d**,**e**) or rank (Mantel–Cox) test (**c**). A sharp symbol (#) indicates that no samples were collected because ferrets in that group died.

Body temperature increased beginning at 4 dpi and peaked at 6 dpi in the IM group. The mean body temperature of ferrets in this group was 40.5 °C (Figure 1a). Furthermore, a continuous decrease in body weight was observed from 4 dpi until death occurred. There was a decrease in body weight of more than 10% in the IM group compared to the body weight of ferrets in the mock group. The mortality rate of ferrets in the IM group was 100% within 10 dpi. Ferrets in the SC group showed similar patterns of temperature and body weight changes and survival rates as those in the IM group; however, the manifestation of clinical symptoms and death among ferrets in the SC group was delayed by 2 days. The mean peak temperature in the SC group was 40.7 °C at 8 dpi with a gradual weight loss of more than 10% of the average until 12 dpi (Figure 1b), when all the infected animals succumbed to the infection (Figure 1c). The body temperature of the animals infected via the IV route rapidly peaked at 41.3 °C at 4 dpi; however, the animals survived and were back to normal at 8 dpi (Figure 1a). Although IV-infected ferrets showed a more rapid decrease in body weight until 8 dpi (~15%, *p* < 0.001), only 25% of ferrets in this group succumbed to the infection by 10 dpi, and the remaining ferrets showed stabilized body weights beginning at 8 dpi (Figure 1b).

### 3.2. Viral Copy Number Changes in Serum and Tissues

To investigate the association between the virulence of SFTSV and viral replication, the viral copy number in blood was quantitated following SFTSV infection (Figure 1d). Ferrets in the IM and SC groups had comparable viral copy numbers during the infection period. The viral copy number gradually increased from 2 dpi and reached a peak titer of 4.7 log_10_/mL at 6 dpi in the IM group. In the SC group, ferrets demonstrated 5.4 log_10_/mL viral copies at 8 dpi and peaked at 5.8 log_10_/mL, albeit with only one sample. In contrast, animals infected through the IV route showed a higher initial viral replication at 2 dpi, compared to animals in the IM or SC group (*p* < 0.0001). The viral copy number in the IV group peaked earlier, at 4 dpi (4.8 log_10_/mL), but gradually decreased until no virus was detected by 10 dpi.

To investigate whether the altered virulence corresponds with viral replication in organs, the viral load was measured in the spleen, liver, kidney, intestine, and brain tissues (Figure 1e). The tissues in the IM and SC groups demonstrated comparable viral copy numbers; however, these groups showed higher viral loads compared with the IV group. Moreover, the spleen and liver showed markedly higher viral loads (3.1–3.7 vs. 1.2–2.3 log_10_ RNA/g), and there were higher viral copy numbers among all other tissues as well. Virus was also detected in the intestine and brain in the IM and SC groups, with 1.1–2 log_10_ RNA/g.

These results indicate that the persistence of viruses in blood and higher viral replication in tissues could induce a failure of viral clearance and cause animals to succumb. Therefore, there is a close association between mortality and virus replication in blood and tissues.

### 3.3. Hematological Changes during the SFTSV Infection

**Figure 2:**
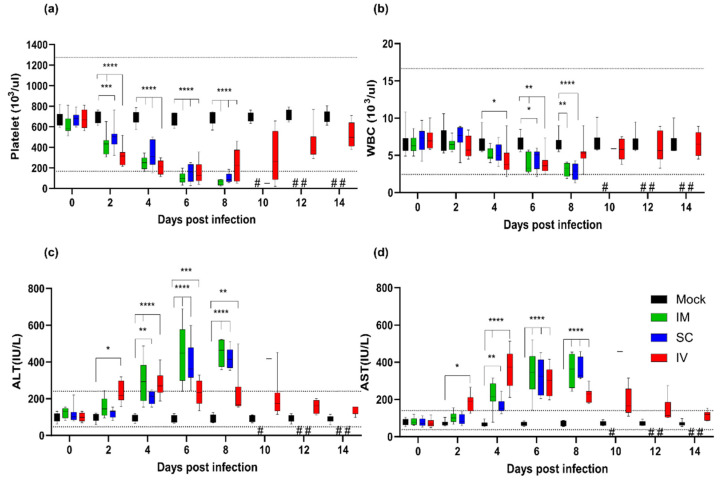
Hematological analysis of aged ferrets after inoculation with SFTSV via the IM, IV, and SC routes. Blood was collected every other day from the IM (green), SC (blue), and IV (red) groups for hematological analysis. (**a**) Platelet count; (**b**) white blood cell (WBC) count; (**c**) alanine aminotransferase (ALT) levels; and (**d**) aspartate aminotransferase (AST) levels. Data are presented as min to max. An asterisk (*) indicates statistical significance when data are compared to those for the mock group by two-way ANOVA with Tukey’s comparison test (* *p* < 0.05; ** *p* < 0.01; *** *p* < 0.001; and **** *p* < 0.0001). A sharp symbol (#) indicates that no samples were collected because ferrets in the group died. The middle part of the dashed line is the normal value of the hematological parameters. Reference values for platelet, WBC, ALT, and AST are 171.7–1280.6 × 10^3^/µL, 2.5–16.7 × 10^3^/µL, 49–242.8U/L, and 40.1–142.7U/L, respectively.

Previous studies have shown that signs of SFTSV infection in aged ferrets include thrombocytopenia, leukopenia, and an increase in liver enzyme levels [8,25,26]. The mean platelet counts gradually decreased and was more than 2-fold lower (247.6 × 10^3^/µL) in the IM group than in the mock group (687.4 × 10^3^/µL) at 4 dpi (Figure 2a). It further decreased significantly to 62.5 × 10^3^/µL at 8 dpi (*p* < 0.0001). Further, they showed a gradual decrease in mean WBC count to a low of 3.1 × 10^3^/µL prior to death (Figure 2b). In addition, mean ALT and AST levels in blood rapidly increased from 4 dpi, reaching 453 and 356.3 IU/L at 8 dpi, respectively (Figure 2c,d). Ferrets in the SC group had similar characteristics to those in the IM group, although they showed a delayed clinical course of infection compared to ferrets in the IM group. In contrast, there was a marked decrease in platelet number from 2 to 8 dpi in the IV group. Surviving ferrets began to recover from thrombocytopenia at 10 dpi; however, there was a continuous decrease in platelet count until death in two animals (Figure 2a). The number of WBCs gradually decreased in the IV group; however, it remained within the normal mean range in most ferrets (Figure 2b). ALT and AST levels increased abnormally until 8 or 10 dpi but returned to their normal ranges from 12 dpi (Figure 2c,d). Taken together, these results indicate that the route of SFTSV infection can affect the mortality rate and disease progression in mice.

### 3.4. Histopathological Tissue Damage Caused by Infection

**Figure 3:**
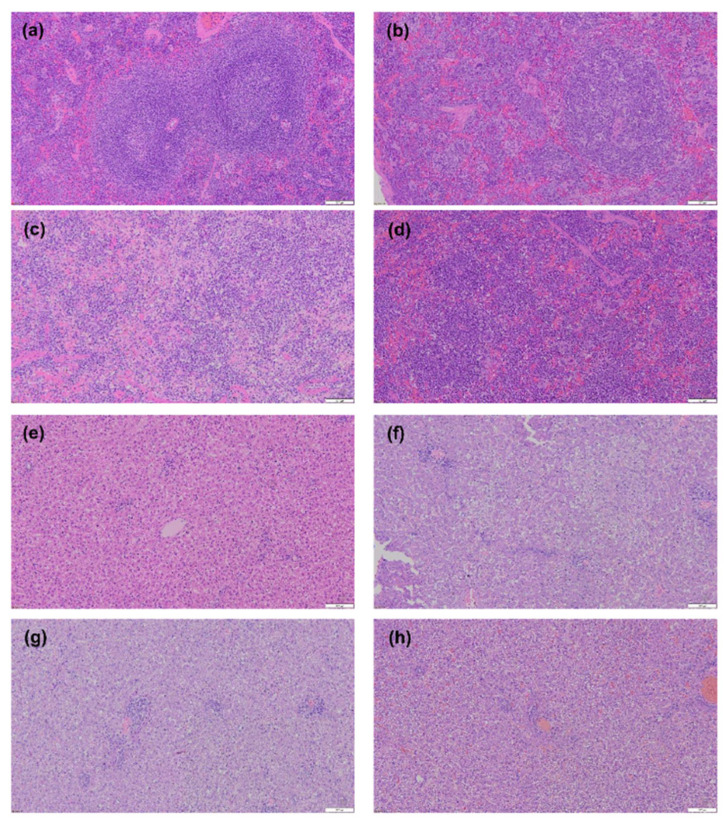
Histopathological observations of tissues from ferrets infected via IM, SC, and IV routes with PBS or 10^5^ FFU SFTSV. The spleen (**a**–**d**) and liver (**e**–**h**) samples were harvested at 6 dpi in the mock (**a**,**e**), IM (**b**,**f**), SC (**c**,**g**), and IV (**d**,**h**) groups. Bars, 100 μm.

To further correlate virulence with pathogenicity, spleen and liver tissues from inoculated ferrets were collected at 6 dpi (Figure 3). All infected groups demonstrated lymphocyte depletion and infiltration of inflammatory cells, while the IM- and SC-infected ferret groups also exhibited loss of splenic white pulp (Figure 3b–d). In contrast, no histopathological changes were detected in uninfected ferrets (Figure 3a). Pathological changes observed in the liver tissues of virus-infected animals included the degradation of hepatocytes, compared with the non-infected sample (Figure 3e–h). Thus, these pathology results are consistent with the massive viral replication seen in these tissues.

## 4. Discussion

SFTSV is a newly emerging zoonotic pathogen in east Asia [2,3,4,5]. However, there is a possibility of its distribution worldwide, as climate change causes the expansion of vector habitats [15,16]. Therefore, studies of vaccines and potential therapeutics for SFTSV infection are ongoing [26,27,28]. However, currently, the most widely used animal model of SFTSV infection is mice deficient in type I IFN signaling (e.g., Ifnar1-/-) [24,29], because adult immunocompetent mice infected with SFTSV have a very low viral replication rate and show few to no signs of infection [20,25]. In contrast, non-human primates exhibit mild clinical signs [19]. Recently, fatal cases of SFTSV infection in a feline model system was reported [30]. In this study, 0.5- to 2-year-old cats were intravenously inoculated with 10^7^ TCID_50_/mL (a higher titer than used in the current study), which resulted in hematological changes with viral replication in body fluids. However, age-dependent differences were not observed in this model. Thus, of the reported animal models, SFTSV-infected ferrets exhibit the most similar response to that of human infection cases, including age-dependent susceptibility and immunocompetency [25].

We found, in our previous study, that aged ferrets infected with SFTSV through the IM route exhibit clinical signs including fever, thrombocytopenia, and liver enzyme changes that are similar to those seen in cases of human infection [25]. However, the results of the animal study suggest that SC injection is better for simulating tick bite-associated infections at similar primary sites of replication prior to viral spread [31]. In contrast, the IV route is not thought to be representative of the natural vector-mediated disease. However, there is increasing concern of infection by transmission via blood transfusion after reports of person-to-person transmission cases through contact with contaminated blood [32]. Therefore, we evaluated each infection route with a time-course analysis of the virological and hematological parameters associated with SFTSV infection.

As a preliminary study, we infected with ferrets with 10^6.2^ FFU to determine whether intravascular infection could also induce virulence, and discovered an 80% survival rate (data not shown). However, we previously reported that IM infection with 10^5^ FFU could induce fatality in infected ferrets [8]. Therefore, we infected aged ferrets with 10^5^ FFU and monitored viral replication and hematological changes. In this study, the protocol included the euthanasia of animals when they exhibited >25% loss of original body weight, neurologic signs, or paralysis. However, the SFTSV-infected ferrets did not meet these criteria, and lethargy was the only clinical sign noted prior to the death of infected animals. Therefore, additional endpoints for the euthanasia of SFTSV-infected animals should be included, such as elevated body temperature (above 41 °C), thrombocytopenia (below 171.7 × 10^3^/µL), and leukopenia (below 2.5 × 10^3^/µL). Moreover, animals exhibiting more than two criteria that persist for two consecutive days should be euthanized to prevent undue suffering [30].

The impact of infection route was explored by varying SFTSV delivery through the IM, SC, and IV routes, and by assessing differences in the mortality rate, virus clearance, tissues titers, hematological changes, and pathology among the groups (Figure 1, Figure 2 and Figure 3). The ferrets in the IM and SC groups showed comparable clinical symptoms and virus replication in serum and tissues, and eventually succumbed to the infection within 12 dpi. In contrast, the SC group showed a delayed clinical outcome. We also observed virally induced tissue damage in the spleen and liver of IM- and SC-infected ferrets. These clinical outcomes and lesions might have been observed as a result of the roles played by resident or recruited immune cells to sites of infection, which could be important for viral entry and infection [33]. After entry at the infection site, SFTSV drains into lymph nodes, where it can target immune cells and impair host immune responses. The virus then enters the systemic circulation and begins the viremia phase of infection. As a consequence, ferrets infected through the IM and SC routes are more likely to succumb to the SFTSV infection.

It is apparent that IV-infected ferrets demonstrate more rapid viral replication and hematological changes than IM- or SC-infected ferrets. In addition, IV-infected ferrets demonstrated viral replication restricted to the spleen, liver, and kidney. Moreover, these animals cleared viruses in blood with only a 25% mortality rate. The succumbed animals demonstrated higher mean peak viral copy numbers in blood than surviving animals (5.8 vs. 4.7 log_10_/mL). Thus, this increased viral replication could increase the risk of fatality. In contrast, the hematological parameters of surviving ferrets returned to normal ranges (Figure 2a).

The lower virulence following IV inoculation could be due to simultaneous antigen presentation in lymph nodes, which might promote faster innate immune responses and clearance of circulating virus. Nevertheless, the virulence of a virus delivered via the IV infection route was shown previously to vary depending on the animal and the virus. Infection of cynomolgus macaques with the CCHF virus through the IV route results in higher pathogenicity compared to injection via the SC route [34]. In contrast, comparable pathogenicity was observed in tick-borne encephalitis virus-infected mice following infection with the virus through the IV and SC routes [35]. It should be noted that viral infection in the gastrointestinal tract might induce gut bacterial leakage into the bloodstream [36]. Therefore, we assessed endotoxin levels in serum harvested at 8 dpi in all groups. However, we could not detect bacterial infection via endotoxin in serum at 8 dpi (data not shown). This might mean that the virulence seen in our study is directly caused by increased viral replication.

Li et al. and Zhao et al. demonstrated that human fatal outcomes are higher in males than in females [6,37]. However, gender differences with respect to virulence in previous animal studies are unclear [19,21,29]. Thus, the possibility of gender differences in animal models should be considered in future studies.

In the present study, our results indicate that virulence and clinical outcomes differ depending on the route of infection. Thus, our results suggest that the most sensitive SFTSV infection route in animal models should be used when investigating the virulence of SFTS, as well as the efficacy of potential antiviral therapies and vaccines. Further investigation of standardized SFTSV infection routes in animals will increase the impact and utility of animal studies. This study suggests that exposure route is an important factor that needs to be taken into consideration when evaluating antiviral regimes and, specifically, for investigating antiviral and vaccine efficacy against SFTSV in the ferret model.

## Data Availability

The data analyzed in this study are included within the paper.

## References

[1] Hu J., Li S., Zhang X., Zhao H., Yang M., Xu L., Li L. (2018). Correlations between clinical features and death in patients with severe fever with thrombocytopenia syndrome. Medicine.

[2] Yu X.-J., Liang M.-F., Zhang S.-Y., Liu Y., Li J.-D., Sun Y.-L., Zhang L., Zhang Q.-F., Popov V.L., Li C. (2011). Fever with thrombocytopenia associated with a novel bunyavirus in China. N. Engl. J. Med..

[3] Kim K.-H., Yi J., Kim G., Choi S.J., Jun K.I., Kim N.-H., Choe P.G., Kim N.-J., Lee J.-K., Oh M.-d. (2013). Severe fever with thrombocytopenia syndrome, South Korea, 2012. Emerg. Infect. Dis..

[4] Takahashi T., Maeda K., Suzuki T., Ishido A., Shigeoka T., Tominaga T., Kamei T., Honda M., Ninomiya D., Sakai T. (2013). The first identification and retrospective study of severe fever with thrombocytopenia syndrome in Japan. J. Infect. Dis..

[5] Tran X.C., Yun Y., Le Van An S.-H.K., Thao N.T.P., Man P.K.C., Yoo J.R., Heo S.T., Cho N.-H., Lee K.H. (2019). Endemic severe fever with thrombocytopenia syndrome, Vietnam. Emerg. Infect. Dis..

[6] Li J.-C., Zhao J., Li H., Fang L.-Q., Liu W. (2022). Epidemiology, clinical characteristics, and treatment of severe fever with thrombocytopenia syndrome. Infect. Med..

[7] Choi S.J., Park S.-W., Bae I.-G., Kim S.-H., Ryu S.Y., Kim H.A., Jang H.-C., Hur J., Jun J.-B., Jung Y. (2016). Severe fever with thrombocytopenia syndrome in South Korea, 2013–2015. PLoS Negl. Trop. Dis..

[8] Yun S.-M., Park S.-J., Kim Y.-I., Park S.-W., Yu M.-A., Kwon H.-I., Kim E.-H., Yu K.-M., Jeong H.W., Ryou J. (2020). Genetic and pathogenic diversity of severe fever with thrombocytopenia syndrome virus (SFTSV) in South Korea. JCI Insight.

[9] Cutler S.J., Vayssier-Taussat M., Estrada-Peña A., Potkonjak A., Mihalca A.D., Zeller H. (2021). Tick-borne diseases and co-infection: Current considerations. Ticks Tick-Borne Dis..

[10] Yamanaka A., Kirino Y., Fujimoto S., Ueda N., Himeji D., Miura M., Sudaryatma P.E., Sato Y., Tanaka H., Mekata H. (2020). Direct transmission of severe fever with thrombocytopenia syndrome virus from domestic cat to veterinary personnel. Emerg. Infect. Dis..

[11] Chen H., Hu K., Zou J., Xiao J. (2013). A cluster of cases of human-to-human transmission caused by severe fever with thrombocytopenia syndrome bunyavirus. Int. J. Infect. Dis..

[12] Yun S.-M., Song B.G., Choi W., Roh J.Y., Lee Y.-J., Park W.I., Han M.G., Ju Y.R., Lee W.-J. (2016). First isolation of severe fever with thrombocytopenia syndrome virus from *Haemaphysalis longicornis* ticks collected in severe fever with thrombocytopenia syndrome outbreak areas in the Republic of Korea. Vector-Borne Zoonotic Dis..

[13] Hu Y.-Y., Zhuang L., Liu K., Sun Y., Dai K., Zhang X.-A., Zhang P.-H., Feng Z.-C., Li H., Liu W. (2020). Role of three tick species in the maintenance and transmission of Severe Fever with Thrombocytopenia Syndrome Virus. PLoS Negl. Trop. Dis.

[14] Han X.-H., Ma Y., Liu H.-Y., Li D., Wang Y., Jiang F.-H., Gao Q.-T., Jiang F., Liu B.-S., Shen G.-S. (2022). Identification of severe fever with thrombocytopenia syndrome virus genotypes in patients and ticks in Liaoning Province, China. Parasites Vectors.

[15] Zhao L., Li J., Cui X., Jia N., Wei J., Xia L., Wang H., Zhou Y., Wang Q., Liu X. (2020). Distribution of *Haemaphysalis longicornis* and associated pathogens: Analysis of pooled data from a China field survey and global published data. Lancet Planet. Health.

[16] Raghavan R., Barker S., Cobos M.E., Barker D., Teo E., Foley D., Nakao R., Lawrence K., Heath A., Peterson A.T. (2019). Potential spatial distribution of the newly introduced long-horned tick, *Haemaphysalis longicornis* in North America. Sci. Rep..

[17] (2018). World Health Organization, 2018 Annual Review of Diseases Prioritized under the Research and Development Blueprint. http://www.who.int/emergencies/diseases/2018prioritization-report.pdf?ua=1.

[18] Tian H., Yu P., Chowell G., Li S., Wei J., Tian H., Lv W., Han Z., Yang J., Huang S. (2017). Severe Fever with Thrombocytopenia Syndrome Virus in Humans, Domesticated Animals, Ticks, and Mosquitoes, Shaanxi Province, China. Am. J. Trop. Med. Hyg..

[19] Jin C., Jiang H., Liang M., Han Y., Gu W., Zhang F., Zhu H., Wu W., Chen T., Li C. (2014). SFTS virus infection in nonhuman primates. J. Infect. Dis..

[20] Matsuno K., Orba Y., Maede-White K., Scott D., Feldmann F., Liang M., Ebihara H. (2017). Animal models of emerging tick-borne phleboviruses: Determining target cells in a lethal model of SFTSV infection. Front. Microbiol..

[21] Jin C., Liang M., Ning J., Gu W., Jiang H., Wu W., Zhang F., Li C., Zhang Q., Zhu H. (2012). Pathogenesis of emerging severe fever with thrombocytopenia syndrome virus in C57/BL6 mouse model. Proc. Natl. Acad. Sci. USA.

[22] Xu S., Jiang N., Nawaz W., Liu B., Zhang F., Liu Y., Wu X., Wu Z. (2021). Infection of humanized mice with a novel phlebovirus presented pathogenic features of severe fever with thrombocytopenia syndrome. PLoS Pathog..

[23] Chen X.-P., Cong M.-L., Li M.-H., Kang Y.-J., Feng Y.-M., Plyusnin A., Xu J., Zhang Y.-Z. (2012). Infection and pathogenesis of Huaiyangshan virus (a novel tick-borne bunyavirus) in laboratory rodents. J. Gen. Virol..

[24] Liu Y., Wu B., Paessler S., Walker D.H., Tesh R.B., Yu X.-j. (2014). The pathogenesis of severe fever with thrombocytopenia syndrome virus infection in alpha/beta interferon knockout mice: Insights into the pathologic mechanisms of a new viral hemorrhagic fever. J. Virol..

[25] Park S.-J., Kim Y.-I., Park A., Kwon H.-I., Kim E.-H., Si Y.-J., Song M.-S., Lee C.-H., Jung K., Shin W.-J. (2019). Ferret animal model of severe fever with thrombocytopenia syndrome phlebovirus for human lethal infection and pathogenesis. Nat. Microbiol..

[26] Kwak J.-E., Kim Y.-I., Park S.-J., Yu M.-A., Kwon H.-I., Eo S., Kim T.-S., Seok J., Choi W.-S., Jeong J.H. (2019). Development of a SFTSV DNA vaccine that confers complete protection against lethal infection in ferrets. Nat. Commun..

[27] Dong F., Li D., Wen D., Li S., Zhao C., Qi Y., Jangra R.K., Wu C., Xia D., Zhang X. (2019). Single dose of a rVSV-based vaccine elicits complete protection against severe fever with thrombocytopenia syndrome virus. npj Vaccines.

[28] Yu K.-M., Park S.-J., Yu M.-A., Kim Y.-I., Choi Y., Jung J.U., Brennan B., Choi Y.K. (2019). Cross-genotype protection of live-attenuated vaccine candidate for severe fever with thrombocytopenia syndrome virus in a ferret model. Proc. Natl. Acad. Sci. USA.

[29] Gowen B.B., Westover J.B., Miao J., Van Wettere A.J., Rigas J.D., Hickerson B.T., Jung K.-H., Li R., Conrad B.L., Nielson S. (2017). Modeling severe fever with thrombocytopenia syndrome virus infection in golden Syrian hamsters: Importance of STAT2 in preventing disease and effective treatment with favipiravir. J. Virol..

[30] Park E.-s., Shimojima M., Nagata N., Ami Y., Yoshikawa T., Iwata-Yoshikawa N., Fukushi S., Watanabe S., Kurosu T., Kataoka M. (2019). Severe fever with thrombocytopenia syndrome phlebovirus causes lethal viral hemorrhagic fever in cats. Sci. Rep..

[31] Zivcec M., Safronetz D., Scott D., Robertson S., Ebihara H., Feldmann H. (2013). Lethal Crimean-Congo hemorrhagic fever virus infection in interferon α/β receptor knockout mice is associated with high viral loads, proinflammatory responses, and coagulopathy. J. Infect. Dis..

[32] Matsumoto C., Shinohara N., Furuta R., Tanishige N., Shimojima M., Matsubayashi K., Nagai T., Tsubaki K., Satake M. (2018). Investigation of antibody to severe fever with thrombocytopenia syndrome virus (SFTSV) in blood samples donated in a SFTS-endemic area in Japan. Vox Sang..

[33] Hofmann H., Li X., Zhang X., Liu W., Kühl A., Kaup F., Soldan S.S., González-Scarano F., Weber F., He Y. (2013). Severe fever with thrombocytopenia virus glycoproteins are targeted by neutralizing antibodies and can use DC-SIGN as a receptor for pH-dependent entry into human and animal cell lines. J. Virol..

[34] Haddock E., Feldmann F., Hawman D.W., Zivcec M., Hanley P.W., Saturday G., Scott D.P., Thomas T., Korva M., Avšič-Županc T. (2018). A cynomolgus macaque model for Crimean–Congo haemorrhagic fever. Nat. Microbiol..

[35] Lieskovská J., Páleníková J., Langhansová H., Chmelař J., Kopecký J. (2018). Saliva of Ixodes ricinus enhances TBE virus replication in dendritic cells by modulation of pro-survival Akt pathway. Virology.

[36] Kim H.S. (2021). Do an altered gut microbiota and an associated leaky gut affect COVID-19 severity?. mBio.

[37] Zhao J., Lu Q.-B., Li H., Yuan Y., Cui N., Yuan C., Zhang X.-A., Yang Z.-D., Ruan S.-M., Liu L.-Z. (2021). Sex Differences in Case Fatality Rate of Patients With Severe Fever With Thrombocytopenia Syndrome. Front. Microbiol..

